# Supplementary material for: Patterns of Intron Gain and Loss in Fungi
Source: PLoS Biol. 2004 Nov 30;2(12):e422. doi: 10.1371/journal.pbio.0020422 (PMC532390; doi:10.1371/journal.pbio.0020422)
Supplement: Table S1 — Also available at http://genes.mit.edu/NielsenEtAl/. (4.3 MB ZIP). [file pbio.0020422.st001.zip › NielsenEtAl/html/171.html]

AN8117.1.NCU06694.1.MG07280.1.FG07347.1


```
 CLUSTAL W (1.82) Multiple Sequence Alignments - Introns Inserted


Sequence 1: NCU06694.1	337 aa
Sequence 2: FG07347.1	343 aa
Sequence 3: MG07280.1	344 aa
Sequence 4: AN8117.1	340 aa
Alignment Length: 349 aa
Number Identitical Residues: 196 aa
Alignment Score (without introns) 9597


MG07280.1 	-----MAEILDKIPMPTIDRPFGVHLWPIFNKAFEAVVGFPADDFHFEVGKTPMSTLKET
NCU06694.1	MATASPVSFLDSLPRPTLDRPFGIHLWPIFSKAFEAVVGYPAEDFRFVPGETPMSTLKET
FG07347.1 	-MSATPATFYEQLPLPTIDRPFGIHLWPIFDKAFTAVVGYSANDFKFVPFETPMSTLKST
AN8117.1  	------MEYLQDIQKPSIERPFGIHLWPIFDKAFEAVMGYPASEFQFVEGKTPMSTFRET
          	          :.:  *:::****:******.*** **:*:.*.:*:*   :*****::.*

MG07280.1 	GIFIVIYYIIIFGGRELMRNREPFKLRSLFLIHNFYLTFISGALLALFIEQLVPTIARRG
NCU06694.1	SIFIVIYYTIIFGGRELMRNREPFKLRTLFLIHNFYLTAISAILLALFIEQLLPIVVRGG
FG07347.1 	SIFIVIYYCIIFGGREWMRNREPFKLKGLFLIHNLYLTLISGTLLALFIEQLLPTVVRGG
AN8117.1  	AIMLIVYYVTIFGGREVMRNRPAFKLNTLFMIHNFVLTAVSAILLALFVEQLVPTIWNHG
          	.*::::**  ****** **** .***. **:***: ** :*. *****:***:* : . *

MG07280.1 	LFFAVCDKEGGWTQPLIVLYY0LNYLTKYLELLDTVFLFLKKKPLT1FLHCYHHGATAFL
NCU06694.1	IFHAICHIDGGWTQPLVVLYY0LNYLTKYLELLDTCFLFLKKKPLT1FLHCYHHGATALL
FG07347.1 	IFHAICDAEGGWTQPLVVLYY0LNYLTKYLELLDTVFLFLKKKPLT1FLHCYHHGATAFL
AN8117.1  	IFYSICDHRGGWTQPLIVLYY0LNYLNKYLEFLDTVFLFLKKKPLT~FLHTYHHGATALL
          	:*.::*.  *******:**** ****.****:*** ********** *** *******:*

MG07280.1 	CYTQLIGSTAVQWSVITLNLMVHVVMYWYYFQSARGIKIWWKEWITRLQITQFVIDLG1F
NCU06694.1	CYTQLIGSTSVSWVPITLNLTVHVVMYWYYFQSARGIRIWWKEWITRLQIIQFIIDLG1F
FG07347.1 	CYTQLIGSTSVSWVPIVLNLLVHVVMYWYYFQSARGVRVWWKEWVTRLQIIQFVIDLG~F
AN8117.1  	CYTQLIGLTAVQWVPITINLLVHVVMYWYYFQSARGIRIWWKKYITRLQIIQFVIDLV1F
          	******* *:*.*  *.:** ***************:::***:::***** **:***  *

MG07280.1 	IYFASYTYFTSTYWPWMPNAGQCAGEEFAAFAGIASISSYLVLFISFYLATYKKERKPPT
NCU06694.1	VYFASYTYFTSTYFPWMPNAGKCAGEEFAAFSGIGILSSYLVLFISFYFATYKKDGKRPT
FG07347.1 	IYFASYTYFTSTYFQWMPNAGKCSGEEFAAFSGIITISSYLVLFISFYFATYKKQGKAPT
AN8117.1  	VYFASYTYFASTYFPWAPNAGNCAGEEFAAFAGMGILTSYLVLFISFYIVTYNKAAK--T
          	:********:***: * ****:*:*******:*:  ::**********:.**:*  *  *

MG07280.1 	ARKSLRRMSQAPLPDPHSLMGAEGAATPSKAANGFSTGAKTNGSSTPRSRKA
NCU06694.1	GRKSLRRMSQAPLPDPKDIVS-------AKASGARPSGAAT-----ARSRKA
FG07347.1 	GRQSLRRMSQAPLPDPHLVQTTP----VKKSNGASTTGAKTN-SASARSRKA
AN8117.1  	GRPRRNTGKQAVIDMARYEVAPPSPAGEKKSNGSAVTTGRSN-GPATRSRKA
          	.*   .  .** :  .:    . ..:   *: ..  : . :. ..:.*****
```
